# Enhancement of Oxygen Mass Transfer and Gas Holdup Using Palm Oil in Stirred Tank Bioreactors with Xanthan Solutions as Simulated Viscous Fermentation Broths

**DOI:** 10.1155/2013/409675

**Published:** 2013-11-17

**Authors:** Suhaila Mohd Sauid, Jagannathan Krishnan, Tan Huey Ling, Murthy V. P. S. Veluri

**Affiliations:** ^1^Faculty of Chemical Engineering, Universiti Teknologi MARA (UiTM), 40450 Shah Alam, Selangor, Malaysia; ^2^Faculty of Chemical Engineering, Manipal International University, 71800 Nilai, Negeri Sembilan Darul Khusus, Malaysia

## Abstract

Volumetric mass transfer coefficient (*k*_*L*_ *a*) is an important parameter in bioreactors handling viscous fermentations such as xanthan gum production, as it affects the reactor performance and productivity. Published literatures showed that adding an organic phase such as hydrocarbons or vegetable oil could increase the *k*_*L*_ *a*. The present study opted for palm oil as the organic phase as it is plentiful in Malaysia. Experiments were carried out to study the effect of viscosity, gas holdup, and *k*_*L*_ *a* on the xanthan solution with different palm oil fractions by varying the agitation rate and aeration rate in a 5 L bench-top bioreactor fitted with twin Rushton turbines. Results showed that 10% (v/v) of palm oil raised the *k*_*L*_ *a* of xanthan solution by 1.5 to 3 folds with the highest *k*_*L*_ *a* value of 84.44 h^−1^. It was also found that palm oil increased the gas holdup and viscosity of the xanthan solution. The *k*_*L*_ *a* values obtained as a function of power input, superficial gas velocity, and palm oil fraction were validated by two different empirical equations. Similarly, the gas holdup obtained as a function of power input and superficial gas velocity was validated by another empirical equation. All correlations were found to fit well with higher determination coefficients.

## 1. Introduction

In aerobic fermentations, the bioreactor performance greatly depends on its oxygen transfer capacities. Oxygen is a soluble substrate, but its solubility in aqueous media at ambient conditions is very low [1]. Thus, actively growing cells can consume all the dissolved oxygen quickly and, hence, oxygen has to be supplied continuously by mass transfer from air to the growth medium [2]. If the oxygen transfer rate to the aqueous phase exceeds the rate of oxygen consumed by the cells, cell growth continues at an exponential rate when other nutrients are not limited. However, when oxygen is not enough, the microorganisms' metabolic rate decreases drastically leading to reduced growth and productivity. 

Xanthan gum is a natural polysaccharide produced by *Xanthomonas campestris* and is an important industrial biopolymer. It is widely used in industries such as foods and cosmetics, pharmaceutical and petroleum industry as emulsion stabilizer, thickener, dispersing agent and drilling fluid, and for many more applications [3]. Xanthan gum is soluble in cold or hot water and its solution can be highly viscous even at low concentration. Xanthan gum solutions show non-Newtonian behavior, that is, pseudoplastic or shear thinning, and the viscosity decreases with increasing shear rate. Besides, the viscosity also depends on its concentration, temperature, concentration of salts, and pH [4]. The fermentations of xanthan gum production are often associated with a significant decrease in oxygen transfer rate because of the increase in viscosity from accumulation of xanthan. As *X. campestris *is a strictly aerobic microorganism, oxygen is an essential nutrient both for growth and for xanthan production. Therefore, oxygen limitation turns into the controlling step in the whole process of xanthan production [5].

There have been various strategies to improve the oxygen transfer in bioreactors. Some of the previous researchers [6–10] adopted an approach of dispersing a nonaqueous, organic, second liquid phase that is immiscible to the system, referred to later as organic phase(s). The presence of organic phase modifies the medium in such a way that it could carry more oxygen and this approach was found successful in the past. The organic phase has strong affinity for oxygen so that it can increase the apparent solubility of oxygen in water [7]. The organic compounds used were hydrocarbons, perfluorocarbons, and vegetable oils.

This method also was applied in xanthan gum fermentation by Ju and Zhao [11] and Kuttuva et al. [12]. They postulated that this method has the ability to solve the viscosity problem and indirectly enhance the oxygen transfer. This was proven by Lo et al. [8] who found that in 3.5% of xanthan solution, the *k*_*L*_ *a* values in the xanthan solution-hexadecane emulsion (0.50 v/v) system were higher than the *k*_*L*_ *a* values obtained in a centrifugal packed bed reactor and stirred tank reactor. While those researchers opted for higher organic phase concentration, this study has focused on the effect of palm oil on the viscosity and the oxygen transfer characteristics in xanthan solution at lower oil concentrations.

In this work, palm oil was chosen as the organic phase to study the oxygen transfer characteristics such as effect of viscosity, gas holdup, and mass transfer coefficient on the xanthan gum solution by varying the agitation rate and aeration rate in a stirred tank bioreactor.

## 2. Materials and Methods

### 2.1. Bioreactor

**Figure 1:**
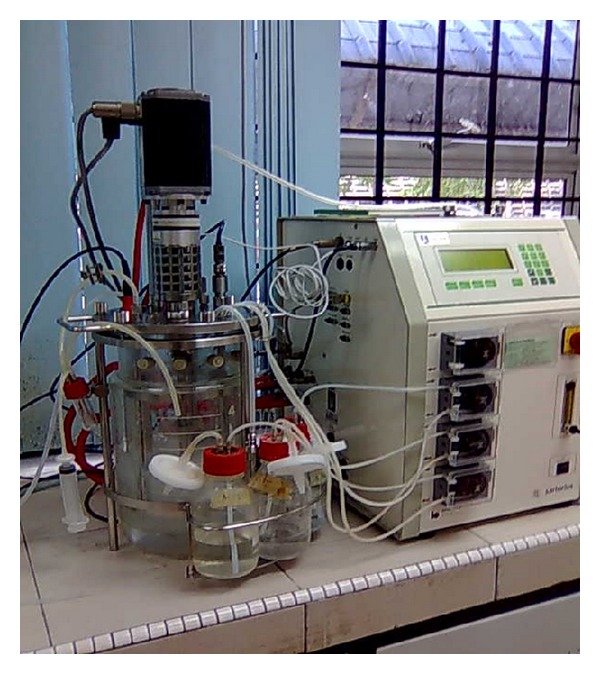
Experimental setup.

Experiments were carried out in an automated 5 L bench-top bioreactor (Biostat B, Sartorius BBI Systems, Germany) with a working volume of 4 L. The height/diameter ratio of glass vessel was 2 : 1. It was equipped with pH electrode (Mettler Toledo, Switzerland), dissolved oxygen (DO) probe (Mettler Toledo, Switzerland), temperature, and antifoam sensor. An overhead stirrer (Heidolph RZR 2102 Control, Germany) with agitation controller and torque reading was mounted on the stirrer shaft on top of the bioreactor for mixing. The bioreactor mixing system was equipped with four baffles and two impellers. Twin Rushton turbine blades spaced 80 mm apart having 64 mm diameter and 13 mm width were used. There was a ring sparger underneath the bottom turbine having of 14 holes 1 mm size each. Standard operating procedure was carried out on each experiment and the DO and pH probes were calibrated before starting bioreactor. For all experiments, the bioreactor was aerated at three different rates, namely, 0.25, 0.75, and 1.25 vvm and agitated at three different speeds, namely, 400, 600, and 800 rpm. The bioreactor used in this study is shown in Figure 1. All the experiments were carried out at atmospheric pressure and the temperature was maintained at 30°C. 

### 2.2. Model Media

The investigation of the effect of palm oil on *k*_*L*_ *a* was conducted in a model media, xanthan gum solutions to represent aqueous solutions of different viscosities as a fermentation broth. In this research, commercial food grade xanthan gum obtained from Bagus Bakery, Malaysia, was used and the xanthan gum solution was prepared at 0.25% (w/v). Xanthan gum was selected as it showed a good non-Newtonian (pseudoplastic) behavior as required besides being inexpensive. 

### 2.3. Palm Oil

Palm oil used in this research was RBD (refined, bleached, and deodorized) palm olein obtained from Alami Technological Services Sdn Bhd, Malaysia. Its viscosity and density were measured as 67.85 cP and 818 kg/m^3^ at the ambient temperature, respectively. The oxygen solubility of palm oil is 47.7 mg/L at 30°C [13] and it has low solubility in water, around 100 mg/L at 28°C [14]. 

### 2.4. Rheology Measurements

The viscosity measurement of xanthan gum solution was conducted using a viscometer (Brookfield LVDV-II+Pro, USA) at 30°C with a SC4-25 spindle. The shear stress versus shear rate data were analyzed as per the Ostwald de Waele or power law model given in (1). In order to study the effect of palm oil dosage, experiments were carried out at different volumetric fractions (0, 5, 10, 15, 20, and 50%) of palm oil in the xanthan solution:

(1) $$\begin{array}{c} \tau =K\gamma^{n}. \end{array}$$

### 2.5. Power Input

The power input was measured by using an overhead stirrer with torquemeter (Heidolph RZR 2102 Control, Germany). The power input was obtained by applying the following:

(2) $$\begin{array}{c} P=2\pi N\left(T-T_{o}\right). \end{array}$$

### 2.6. Probe Response Time of DO Meter

In determining *k*_*L*_ *a* values, it is important to find out the probe response time. Response time, *τ*_*r*_, is defined as the time needed to record 63% of the final value measured when exposed to a stepwise change of concentration [15]. The probe response time was determined by transferring the dissolved oxygen probe (InPro 6820 Series, Mettler Toledo, Switzerland) from oxygen-free solution (0% saturation value) to an oxygen saturated solution (100% saturation value). The abrupt rise in the DO reading from 0% to 100% was monitored and recorded every five seconds. Then, the probe response was modeled as first-order dynamic as described by García-Ochoa et al. [5] in the following:

(3) $$\begin{array}{c} \frac{dC_{\text{me}}}{dt}=\frac{C_{L}-C_{\text{me}}}{\tau_{r}}. \end{array}$$

Upon linearization, (3) became

(4) $$\begin{array}{c} \ln\frac{C_{L}-C_{\text{me}}}{C_{L}-C_{\text{m}{\text{e}}_{o}}}=-\frac{t}{\tau_{r}}. \end{array}$$

A plot of ln⁡(*C*_*L*_ − *C*_me_/*C*_*L*_ − *C*_me_*o*__) against *t* yielded a straight line with inverse slope value of 1/*τ*_*r*_. From that the probe response time obtained was found to be 24.16 s.

### 2.7. Volumetric Mass Transfer Coefficient

For the *k*_*L*_ *a* value determination the static gassing out method was employed throughout the experiments. This was performed by firstly purging the system (xanthan solution with palm oil) with nitrogen gas until the dissolved oxygen fell to zero. When DO was stabilized at 0% value, the nitrogen valve was switched off and, simultaneously, the aeration was started and time was marked as zero (*t* = 0). The gradual increase of DO concentration was monitored and recorded until it reached a steady value at 100%. In order to calculate the *k*_*L*_ *a* value, (5) was used:

(5) $$\begin{array}{c} \frac{dC_{L}}{dt}=k_{L}a\left(C_{L}^{*}-C_{L}\right) \end{array}$$

which on integration yielded

(6) $$\begin{array}{c} \ln\left(1-\frac{C}{C^{*}}\right)=-k_{L}a\cdot t. \end{array}$$

The *k*_*L*_ *a* value was determined from the slope of plot ln⁡(1 − *C*_*L*_/*C*_L_*) versus *t* where *C*_L_* is the equilibrium dissolved oxygen concentration. The effect of the probe response was neglected as the time required for the oxygen transfer 1/*k*_*L*_ *a* was high compared to the dynamic response of the probe (1/*k*_*L*_ *a* ≫ 10*τ*_*r*_) [12]. However, in case of the slower response, the dynamic response of the probe must be taken into account for the calculation of *k*_*L*_ *a* [15–17]. Considering the effect of response time of the DO probe used, the *k*_*L*_ *a* value obtained from (6) served only as the initial guess value to compute the actual *k*_*L*_ *a* value. Combining (3) and (5) yielded a nonlinear regression (7) for the experimental DO data which was later solved numerically to find the actual *k*_*L*_ *a* value. Consider

(7) $$\begin{array}{c} C_{\text{me}}=C^{*}+\frac{C^{*}-C_{\text{m}{\text{e}}_{o}}}{1-\tau_{r}k_{L}a}\left[\tau_{r}k_{L}a\exp\left(-\frac{t}{\tau_{r}}\right)-\exp\left(-k_{L}a\cdot t\right)\right]. \end{array}$$

### 2.8. Gas Holdup

The gas holdup of the system was measured by the difference between the average liquid level with and without aeration using (8) given below. Consider

(8) $$\begin{array}{c} \varepsilon_{G}=\frac{H_{G+L}-H_{L}}{H_{G+L}}. \end{array}$$

### 2.9. Empirical Correlations

There are several empirical correlations found in the published literature to predict *k*_*L*_ *a* values; however, the one developed by Cooper et al. [18] is widely used. It relates *k*_*L*_ *a* to the specific power input and the superficial gas velocity as given in the following:

(9) $$\begin{array}{c} k_{L}a=\delta {\left(\frac{P_{g}}{V_{L}}\right)}^{\beta }v_{s}^{\alpha }. \end{array}$$

The power input term in the correlation includes all the effects of flow and turbulence on bubble dispersion and the mass transfer boundary layer according to Doran [1]. The values of the constants may vary depending on the system's geometry, the range of variables investigated, and the experimental technique applied. The range of values of *β* and *α* for Newtonian fluids varied between 0.4 to 0.95 and 0.2 to 0.75, respectively [19]. 

However, Nielsen et al. [20] modified (9) to include the organic phase term as in (10), where (*P*_*g*_/*V*_*L*_) represents the power input term, *v*_*s*_ represents the superficial gas velocity term, and *ϕ*_ORG_ represents the palm oil fraction term. The symbols of *δ*, *β*, *α*, and *γ* represent the empirical constants to be determined. Consider

(10) $$\begin{array}{c} k_{L}a=\delta {\left(\frac{P_{g}}{V_{L}}\right)}^{\beta }v_{s}^{\alpha }{\left(1-\phi_{\text{ORG}}\right)}^{\gamma }. \end{array}$$

Gas holdup, *ε*_*G*_, generally depends on superficial gas velocity, power consumption, surface tension and viscosity of liquids and solid concentration [15]. For an agitated reactor, the most common gas holdup correlation used is as shown by (11) [21–23]. The symbols *χ*, *λ*, and *ω* represent empirical constants specific to the system under investigation. Consider

(11) $$\begin{array}{c} \varepsilon_{G}=\chi {\left(\frac{P_{g}}{V_{L}}\right)}^{\lambda }v_{s}^{\omega }. \end{array}$$

## 3. Results and Discussion

The presence of palm oil in xanthan solution resulted in significant changes in the viscosity and rheological behavior, volumetric mass transfer coefficient, and gas holdup with respect to the different values of palm oil volume fraction, agitation rate, and aeration rate. The results obtained are discussed as follows.

### 3.1. Effect of Palm Oil on the Viscosity and Rheology of Xanthan Gum Solution

Xanthan solution (0.25%, w/v) exhibited a non-Newtonian pseudoplastic behavior. As palm oil was added into the xanthan solution at different oil fractions as presented in the Table 1, the rheological characteristics were changed. It was observed that the addition of palm oil slightly reduced the value of *n* and increased the value of *K* gradually in the power law (1). As the *n* value reduced with increase in the palm oil fractions, the degree of the pseudoplasticity of the xanthan solution increased with the increase in palm oil fraction than the pure xanthan solution.

**Figure 2:**
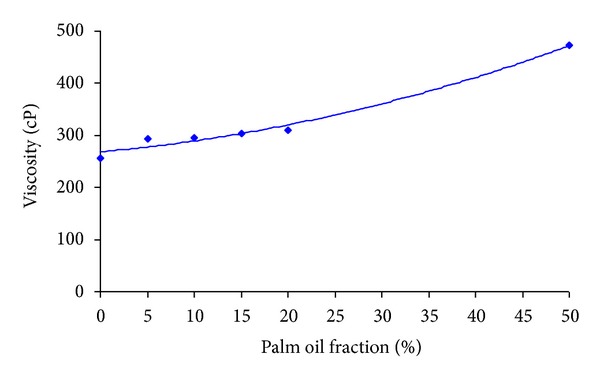
Variation of apparent viscosity of xanthan solution with palm oil fraction at a shear rate of 12.56 s^−1^.

 The viscosities of the xanthan solution with different palm oil fractions were measured as a function of the shear rate and the trend showed that, for all palm oil fractions, the viscosity decreased with the increase in shear rate and reached almost plateau at the higher shear rates. This trend showed that the solutions were in good agreement with the pseudoplastic nature of the xanthan solution. The apparent viscosity of the solutions at different palm oil fractions at a shear rate of 12.56 s^−1^ was illustrated in Figure 2. It showed that the viscosity of xanthan solution increased gradually as the palm oil fraction was increased. Similar trend was obtained by Ma and Babosa-Cánovas [24] and they explained that, since the mean distance between the droplets was smaller, it facilitated compacting of oil, thereby leading to increase in the viscosity.

### 3.2. Effect of Palm Oil on *k*_*L*_ *a* in Xanthan Gum Solution

**Figure 3:**
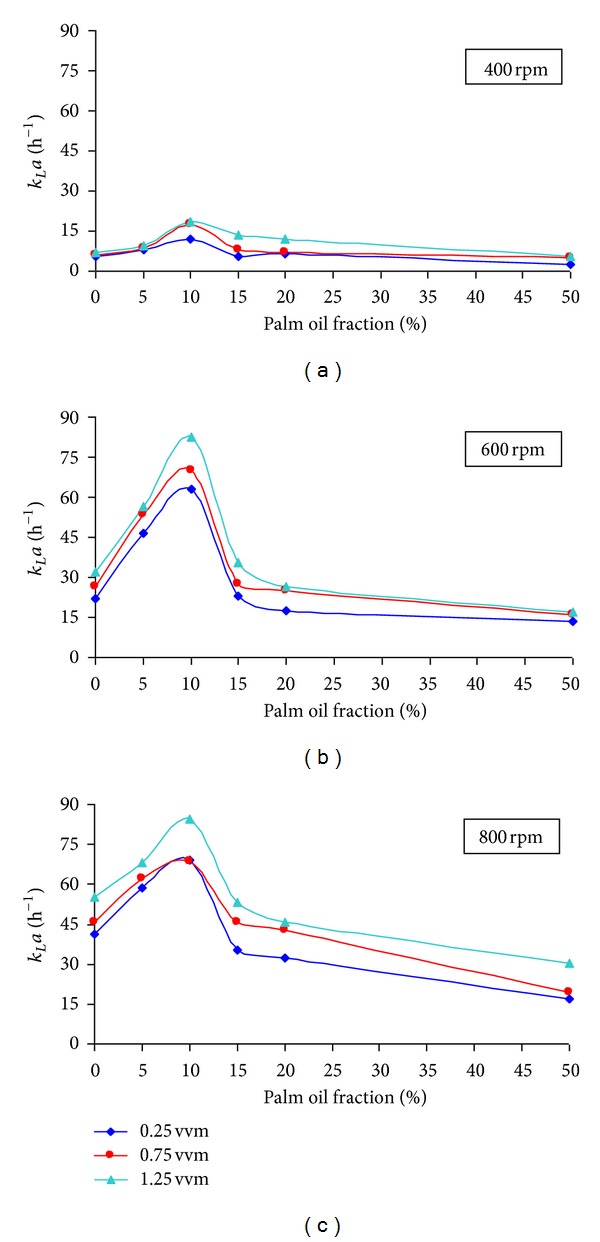
Influence of palm oil on *k*_*L*_ *a* at varying agitation and aeration rate.

Figure 3 showed the effect of palm oil on *k*_*L*_ *a* as a function of agitation and aeration rate at different palm oil fraction. Comparing the values of *k*_*L*_ *a* at the agitation rates of 400, 600, and 800 rpm, respectively, it was observed that the *k*_*L*_ *a* values increased with the increase in agitation rate. Similar trends were also observed when the aeration rate was increased. The increase in aeration and agitation rate increased the degree of the liquid turbulence in the bioreactor. The liquid turbulence created was favorable to increase the *k*_*L*_ *a* as it reduced the liquid film thickness at the gas-liquid interface [25]. Furthermore, the agitation was responsible for producing smaller bubbles, thus increasing the interfacial area, *a*, and it also increased the residence time of air bubbles [15, 25]. As a consequence, higher *k*_*L*_ *a* with the increasing aeration and agitation rate was observed.

From the Figure 2, it is clearly showed that *k*_*L*_ *a* was enhanced in the presence of palm oil. It was found that *k*_*L*_ *a* was higher at 10% of oil fraction than that of pure xanthan solution for all the aeration and agitation rates. The highest *k*_*L*_ *a*, 84.44 h^−1^, was obtained at 1.25 vvm and 800 rpm. Most importantly, *k*_*L*_ *a* values obtained for 10% oil fraction at 600 rpm were almost similar to that of 800 rpm. This observation proved that with the presence of 10% of palm oil in the xanthan solution, high oxygen transfer could be obtained without supplying any additional energy. 

Rols et al. [26] suggested a few possible reasons for the increments in *k*_*L*_ *a* with the addition of organic phases to the fermentation broth. The increments in *k*_*L*_ *a* was resulted from the increase in the liquid turbulence contributed from the rigid organic phase droplets. In addition to that the organic phase formed a thin layer at the gas-liquid interface acting as intermediary for transport of oxygen to the aqueous phase. 

Further, as suggested by Yoshida et al., [6] the spreading coefficient value, *S*, also plays a major role in altering the oxygen transfer capability of the system. For an organic phase having a negative value of spreading coefficient would form into floating lens like droplets in the system, while the coefficient being positive, the organic phase would spread on the water surface like a surface active agent to lower the surface tension and thereby increase the interfacial area, *a*, ultimately increasing *k*_*L*_ *a*. Since the palm oil used in the present work also has a positive value of spreading coefficient (*S* = 38.3 mN/m), it is evident that the surface tension of the xanthan solution was reduced by the palm oil and hence decreased the air bubble size leading to increased interfacial area and *k*_*L*_ *a*. Similar results were reported by Yoshida et al. [6] for toluene in water and oleic acid in water system with toluene and oleic acid having positive values of spreading coefficient.

The enhancements of *k*_*L*_ *a* at 10% of oil fraction were more evident at 400 rpm and 600 rpm than the enhancements at 800 rpm. This could be seen in the form of enhancement factor as shown in Table 2. Enhancement factor can be defined as the ratio of *k*_*L*_ *a* value in xanthan solution with palm oil, (*k*_*L*_ *a*)_po_ to that of pure xanthan solution, (*k*_*L*_ *a*)_*o*_ both measured at the same aeration and agitation rate. In this table, it could be observed that *k*_*L*_ *a* was enhanced by almost three times at 400 rpm and 600 rpm compared to that of 800 rpm as the enhancement factor was only 1.57 in average. 

The enhancement factor decreased with increase in the aeration and agitation rate. Similar trend was also observed by Galaction et al. [9] for the dodecane as the organic phase in the fermentation broth. They justified that reduction in the enhancement factor was due to the disruption of the dodecane superficial film or the removal of dodecane droplets from the bubble surface caused by the intensification of the mixing and turbulence at higher aeration and agitation rate. However, in this study, when the agitation rate was increased to 800 rpm, the mixing was intense and with further addition of the palm oil yielded tiny bubbles, thereby lowering the value of *k*_*L*_ [1] which contributed to the lower enhancement factor at 800 rpm compared to that of 400 and 600 rpm.

However, further increase in the oil fractions was found to decrease the *k*_*L*_ *a* values. With the increase in palm oil fraction, the viscosity as well as the degree of the pseudoplasticity was found to increase as shown in Table 1. Small bubbles that produced in such solution remained in the solutions due to reduced rising velocity. They became rigid spheres having lower *k*_*L*_ value due to surface immobility and no gas circulation [17, 28]. Therefore, it is concluded that the effect of the increase in viscosity and the change in rheology of the solution with the increase in palm oil fraction outweighed the effect of decreased surface tension, which resulted in the decrease of *k*_*L*_ *a*. 

### 3.3. Effect of Palm Oil on Gas Holdup in Xanthan Gum Solution

**Figure 4:**
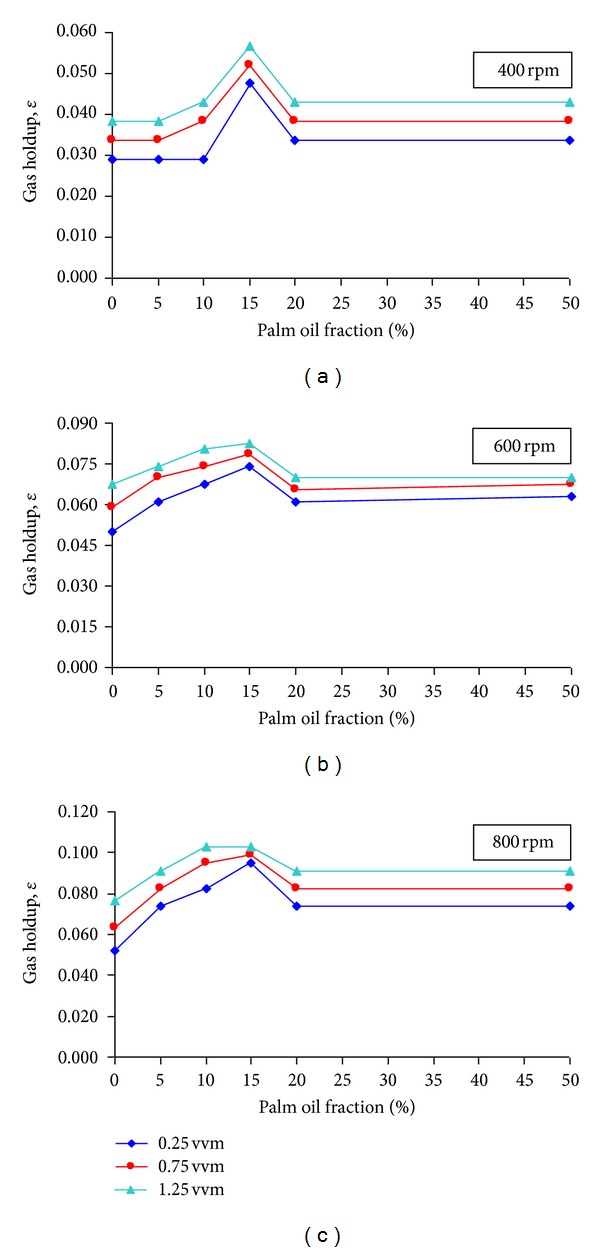
Influence of palm oil on gas holdup at varying agitation and aeration rate.

As seen in Figure 4, the effect of palm oil on gas holdup in the xanthan solution showed similar trend for all the ranges of agitation and aeration rate studied. The gas holdup increased with the increase in palm oil fraction up to 15%. The increase in the gas holdup with the palm oil addition could be due to the dispersion of small bubbles in the system. As discussed earlier in Section 3.2, palm oil might have spread on the bubble surface, thus reducing the surface tension and decreasing the bubble size. The smaller bubbles had induced the gas holdup due to their low rise velocity and longer residence time in the system than the bigger bubbles [1, 29]. This result is in agreement with Kawase and Moo-Young [30] who also found increments in gas holdup in their CMC solution with antifoam addition. They postulated that antifoam promoted the bubble breakup rather than bubble coalescence. 

However, when the palm oil fraction reached beyond 20%, the gas holdup decreased and maintained the same until 50%. As mentioned in Section 3.1, palm oil addition into the xanthan solution changed the viscosity and the degree of pseudoplasticity of the solution. According to Machon et al. [31], the increase in the degree of pseudoplasticity had affected the stirrer's ability to dissipate power to the system to create smaller bubbles when the sparged air passed through and into the stirrer region. In addition, when the viscosity of the system increased, the degree of the liquid turbulence decreased which induced the bubble coalescence and these occurrences increased the proportion of larger bubbles in the liquid [32, 33] which had high rise velocities raced to the surface and ultimately reduced the gas holdup. Furthermore, even though the gas holdups at these fractions (20% and 50%) were lower than at the rest of the oil fraction, they were still higher than the holdup in pure xanthan solution. Hence, some of the tiny bubbles tend to remain lodged in the solution which contributed to the higher gas holdup. Similar occurrences were also observed by Doran [1]. 

### 3.4. Correlations for Volumetric Mass Transfer Coefficient in Xanthan Gum Solution

This experimental study is the first of its kind to report the correlations for *k*_*L*_ *a* obtained for palm oil in xanthan solution. Other organic phases were used in various fermentation broths and for a few bioreactor systems. Equation (10) was used to fit the correlations for *k*_*L*_ *a* in the literature which are listed in Table 3. whereas in this study, the experimental data was fitted into both correlations (9) and (10) to find *k*_*L*_ *a* for palm oil in xanthan solution. Equation (9) considered the effect of superficial gas velocity and power input on *k*_*L*_ *a* while (10) took into account the effect of palm oil fraction in addition. The experimental data was fitted well into both equations with high correlation coefficients. The values of the empirical constants obtained are listed in Table 3. 

While using (9), the *k*_*L*_ *a* values obtained were highly dependent on the specific power input supplied and the empirical constants obtained were within the range suggested by Winkler [19]. This indicated that a change in specific power input would change the *k*_*L*_ *a* significantly than the change in the superficial gas velocity. However, while using (10), the exponent of palm oil fraction dominated the correlation. This meant that the oil fraction had the highest influence on *k*_*L*_ *a*. The negative sign at the exponent value showed that adding palm oil up to 10% volume fraction would increase the *k*_*L*_ *a*. 

According to Kawase and Moo-Young [34], for a non-Newtonian system, the suggested values of *β* are between 0.37 and 0.80, while, for *α*, the values are between 0.20 and 0.84. Comparing the values in Table 3, it was found that *β* values obtained in this study were within the range specified by Kawase and Moo-Young [34]. However, for *α*, the values were found lower at 5% and 10% oil fractions, respectively. As this constant is a measure of aeration rate, it corresponds to lower *k*_*L*_ *a* values compared to that of other oil fractions. It might be due to insufficient aeration compared to the other oil fraction. However, the constant *γ* was not comparable. This could be due to the difference in the bioreactor geometry, type of organic phase used in the study, and different rheological behavior of the bioreactor systems.

### 3.5. Correlations for Gas Holdup in Xanthan Gum Solution

Again this study is the first of its kind to report the correlation for *ε*_*G*_ obtained for palm oil in xanthan solution. Few researchers used (11) for their agitated vessels handling different solution, to fit the correlation for *ε*_*G*_ which are listed in Table 4.

The correlations found in the literature considered only the empirical constants *λ* and *ω* in (11), whereas in this study all the three terms were considered and the results are listed in Table 4. For xanthan solution, the empirical constants ranged within 0.0061 < *χ* < 0.0178, 0.1890 < *λ* < 0.3528, and 0.0773 < *ω* < 0.2180. As the value of *λ* was more than the other two constants for all palm oil fractions, it could be concluded that the change in specific power input had more impact on gas holdup than the change in superficial gas velocity for xanthan solution. 

As shown in Table 4, it can be seen that the *λ* values obtained in this study are comparable with the value obtained by de Figueiredo and Calderbank [21]. However, due to the significant difference in the type and properties such as viscosity and rheological behavior of the organic phase used, a slight variation in the values of *λ* and *ω* is observed when compared to the literature. 

## 4. Conclusions

The volumetric mass transfer coefficient, *k*_*L*_ *a*, of xanthan solution was measured at varying operating variables and palm oil fraction in a stirred tank bioreactor. It was evident that the addition of palm oil up to 10% volume fraction enhanced *k*_*L*_ *a* by 1.5 to 3 folds with the highest *k*_*L*_ *a* value of 84.44 h^−1^. It was also found that increase in palm oil fraction increased the viscosity and rheology of the xanthan solution. This favorable effect was contributed from the properties of palm oil that promoted the production of small bubbles. This ultimately outweighed the positive effect on bubble size and therefore affected both *k*_*L*_ *a* and gas holdup. The *k*_*L*_ *a* values obtained were also correlated with the power input, superficial gas velocity, and palm oil fraction in two different forms of equations which were found to fit well with very high correlation coefficients. 

## Figures and Tables

**Table 1:** Rheological characteristics of xanthan solution at different palm oil fractions.

Palm oil volume fraction (%)	Flow index, *n *	Consistency index, *K* (*c*P^*n*^)
0	0.4243	1108
5	0.4228	1191
10	0.4217	1280
15	0.4214	1334
20	0.4009	1399
50	0.344	2526

**Table 2:** The enhancement factor of *k*_*L*_ *a* at 10% palm oil fraction.

Agitation rate	Aeration rate		
	0.25 vvm	0.75 vvm	1.25 vvm
	(*k*_*L*_ *a*)_po_/(*k*_*L*_ *a*)_*o*_	(*k*_*L*_ *a*)_po_/(*k*_*L*_ *a*)_*o*_	(*k*_*L*_ *a*)_po_/(*k*_*L*_ *a*)_*o*_
400 rpm	2.23	2.95	2.71
600 rpm	2.85	2.65	2.59
800 rpm	1.68	1.49	1.52

**Table 3:** The empirical constants for *k*_*L*_ *a* reported in the literature and in the current study.

Reference	Liquid system	Type of impeller	Constant *β*	Constant *α*	Constant *γ*	Valid for *P*_*g*_/*V*_*L*_ (kW/m^3^)	*R* ^2^	Average error, %	Correlation
[10]	Perfluorocarbon (PFC) in YPD medium with inactive cells	Rushton turbine	0.302	0.699	−1.378	NA	NA	15.7	Nielsen et al. [20] (10)
[20]	n-Hexadecane in broth medium	Rushton turbine	0.31	0.70	1.70	NA	NA	NA	
[27]	Methyl ricinoleate in *Yarrowia lipolytica* broth	Rushton turbine	0.6	0.8	−22	NA	NA	NA	
[27]	Tween 80 in *Yarrowia lipolytica* broth	Rushton turbine	0.4	0.7	−449	NA	NA	NA	

Current study	Xanthan gum solution with 0 to 10% palm oil fraction	Rushton turbine	0.4773	0.1620	−6.6029	0.079 to 1.11	0.77	58.75	Nielsen et al. [20] (10)
	Xanthan gum solution with 0% palm oil fraction	Rushton turbine	0.6674	0.2076	−6.6029	0.141 to 1.11	0.89	46.55	

Current study	Xanthan gum solution with 5% palm oil fraction	Rushton turbine	0.5361	0.1225	−6.1622	0.094 to 0.89	0.78	58.74	
	Xanthan gum solution with 10% palm oil fraction	Rushton turbine	0.4078	0.1702	−8.2896	0.079 to 0.89	0.68	46.47	
	Xanthan gum solution with 15% palm oil fraction	Rushton turbine	0.4401	0.4253	−2.7865	0.058 to 0.83	0.74	40.93	Nielsen et al. [20] (10)
	Xanthan gum solution with 20% palm oil fraction	Rushton turbine	0.4885	0.2777	−1.6270	0.039 to 0.72	0.98	15.59	
	Xanthan gum solution with 50% palm oil fraction	Rushton turbine	0.4334	0.1605	−0.0697	0.031 to 0.64	0.86	34.40	

Current study	Xanthan gum solution with 0% palm oil fraction	Rushton turbine	0.6674	0.2076	—	0.141 to 1.11	0.89	46.55	Cooper et al. [18] (9)
	Xanthan gum solution with 5% palm oil fraction	Rushton turbine	0.5361	0.1225	—	0.094 to 0.89	0.78	58.73	
	Xanthan gum solution with 10% palm oil fraction	Rushton turbine	0.4078	0.1702	—	0.079 to 0.89	0.68	46.47	
	Xanthan gum solution with 15% palm oil fraction	Rushton turbine	0.5343	0.3054	—	0.058 to 0.83	0.96	18.80	
	Xanthan gum solution with 20% palm oil fraction	Rushton turbine	0.4994	0.2740	—	0.039 to 0.72	0.98	14.00	
	Xanthan gum solution with 50% palm oil fraction	Rushton turbine	0.4458	0.3391	—	0.031 to 0.64	0.91	27.67	

**Table 4:** The empirical constants for ε_*G*_ by (11) reported in the literature and in the current study.

Reference	Liquid system	Constant **χ**	Constant **λ**	Constant **ω**	Type of impeller	*R* ^2^	Average relative error (%)	Valid for *P*_*g*_/*V*_*L*_ (kW/m^3^)
[21]	Water	—	0.25	0.75	Flat blade turbine	NA	NA	NA
[22]	Na_2_SO_4_	—	0.4903	0.5788	Rushton turbine	NA	9.3	NA
[23]	Tap water	—	0.478	0.4910	Disc turbine-pitched blade	0.98	NA	NA
[23]	Tap water	—	0.4244	0.6904	Rushton turbine	0.98	NA	NA

Current study	Xanthan gum solution with 0% palm oil fraction	0.0080	0.2385	0.2180	Rushton turbine	0.74	14.47	0.141–1.11
	Xanthan gum solution with 5% palm oil fraction	0.0061	0.3501	0.1435	Rushton turbine	0.90	12.15	0.094–0.89
	Xanthan gum solution with 10% palm oil fraction	0.0065	0.3528	0.1626	Rushton turbine	0.93	10.87	0.079–0.89
	Xanthan gum solution with 15% palm oil fraction	0.0161	0.2507	0.0773	Rushton turbine	0.98	3.27	0.058–0.83
	Xanthan gum solution with 20% palm oil fraction	0.0119	0.2558	0.1395	Rushton turbine	0.96	5.99	0.039–0.72
	Xanthan gum solution with 50% palm oil fraction	0.0178	0.1890	0.1690	Rushton turbine	0.84	12.06	0.031–0.64

## Abbreviations

*a*: Gas liquid interfacial area per unit volume (m^2^/m^3^)
*C*_*L*_*: Oxygen concentration in the equilibrium with the gas phase (mg/L), also known as the solubility (at the phase boundary)
*C*_*L*_: Actual dissolved oxygen concentration in the liquid (mg/L)
CMC: Carboxymethyl cellulose
DO: Dissolved oxygen
*C*_me_: DO concentration in the liquid measured by the probe (mg/L)
*H*_*G*+*L*_: Level of aerated liquid (cm)
*H*_*L*_: Level of unaerated liquid (cm)
*K*: Consistency index (Pa·s^*n*^)
*k*_*L*_: Liquid phase oxygen transfer coefficient (m/s)
*k*_*L*_*a*: Volumetric mass transfer coefficient (h^1^)
*n*: Flow behavior index (dimensionless)
*N*: Impeller speed, s^1^
*P*: Power input, W
(*P*_*g*_/*V*_*L*_): Specific power input in an aerated condition, W/m^3^
*T*: Working torque, N·m
*T*_*o*_: Blank torque, N·m
*S*: Spreading coefficient, mN/m
*ε*_*G*_: Gas holdup
*τ*: Shear stress (N/m^2^)
*γ*: Shear rate (s^1^)
*τ*_*r*_: Response time (s)
*v*_*s*_: Superficial gas velocity, m/s
vvm: Gas volume flow per liquid volume per minute (volume per volume per minute)
*ϕ*_ORG_: Oil fraction in the bioreactor
*δ*, *β*, *α*, *γ*: Empirical constants for *k*_*L*_ *a*, specific to the system under investigation
*χ*, *λ*, *ω*: Empirical constants for *ε*_*g*_, specific to the system under investigation.
